# Diagnostic modalities in vascularized composite allotransplantation: from histopathology to multimodal strategies

**DOI:** 10.3389/fimmu.2026.1804957

**Published:** 2026-05-28

**Authors:** William J. Crisler, Felix J. Klimitz, Samuel J. Steuart, Christine J. Ko, Rachael A. Clark, Bohdan Pomahac, Martin Kauke-Navarro

**Affiliations:** 1 Department of Dermatology, Brigham and Women’s Hospital, Harvard Medical School, Boston, MA, United States; 2 Department of Surgery, Division of Plastic Surgery, Yale School of Medicine, New Haven, CT, United States; 3 Departments of Dermatology and Pathology, Yale School of Medicine, New Haven, CT, United States

**Keywords:** diagnosis, face transplant, mucosa, skin, VCA

## Abstract

**Background:**

Vascularized composite allotransplantation (VCA) of the face and limbs restores form and function after devastating injury but, as in other transplant settings, remains limited by the precision of its diagnostic tools. Current surveillance relies on skin biopsy histology, which addresses acute cellular but not antibody-mediated or chronic rejection. More than a decade of clinical experience has revealed persistent diagnostic gaps, including sampling bias and interobserver variability.

**Methods:**

Published studies describing histopathologic, molecular, and imaging approaches in human VCA were reviewed to summarize diagnostic advances and identify emerging tools.

**Results:**

The Banff 2022 revision introduced vascular modifiers to capture chronic vascular injury. Comparative work indicates that mucosal rejection may occur earlier or more severely than cutaneous rejection. Molecular assays such as donor-derived cell-free DNA, miRNA profiling, and proteomic and transcriptomic panels show promise for early and minimally invasive detection.

**Discussion:**

Key challenges in VCA diagnostics include variability in biopsy interpretation across centers, absence of validated molecular and biomarker criteria, and limited integration of multimodal data into clinical workflows. Coordinated multicenter efforts are needed to standardize evaluation and improve early, accurate diagnosis.

## Introduction

Vascularized composite allotransplantation (VCA) of the face and limbs restores function and appearance after severe injury but remains constrained by the diagnostic tools currently available for rejection monitoring and diagnosis. Long-term graft survival depends on early recognition of immune-mediated injury, and current assessment relies on Banff-based skin biopsy histopathology. This system focuses on acute cellular rejection and does not yet address antibody-mediated or chronic forms, which were not yet definable from the available cases at the time. Histology alone may not capture tissue-specific immune responses, and potential differences among skin, mucosa, and sentinel flaps add diagnostic variability. Nonetheless, the Banff classification — both the 2007 framework and the 2022 update — remains the gold standard and a foundational pillar guiding the diagnosis and management of VCA rejection.

This Mini Review summarizes current diagnostic strategies in human VCA. It also discusses recent updates to the Banff classification and the path toward standardized, multicenter approaches that may refine diagnostic precision and long-term monitoring.

## Histopathology and tissue-based diagnostics

The 2007 Banff working classification is the most validated foundation for histopathological diagnosis of rejection in skin-containing VCA and remains the gold-standard (1). At the time of its description, 41 VCA recipients (three face, 28 hand, nine abdominal wall, one knee with skin island) had been reported (1–4). The grading system describes different grades of T cell-mediated rejection but does not yet include assessment of other tissues (e.g. mucosa), antibody-mediated and chronic rejection because too few cases were available at the time. Unlike limb VCA, most facial allografts include oral and nasal mucosa, and consideration of immune activity at these additional epithelial sites may improve overall assessment of fVCA rejection. Histologic assessment of skin biopsies alone has drawbacks: the procedure is invasive, can cause scarring, and is prone to sampling bias arising from nonrandom site selection, which may inaccurately represent disease severity and distribution. Although mucosal biopsies share similar site-selection limitations, they offer the advantage of being obtained from internal surfaces that typically heal without visible scarring. Evaluating a second epithelial surface alongside skin may therefore provide a more comprehensive view of rejection activity across the allograft, particularly when rejection is patchy or discordant between graft tissues (4, 5).

Distinguishing mild from moderate inflammation is subjective, and interobserver variability limits consistency (6). Despite some limitations the Banff classification remains the gold-standard in assessing fVCA rejection and survey respondents at the 2022 Banff meeting rated the original Banff criteria as important for assessing VCA rejection (7).

**Figure 1:**
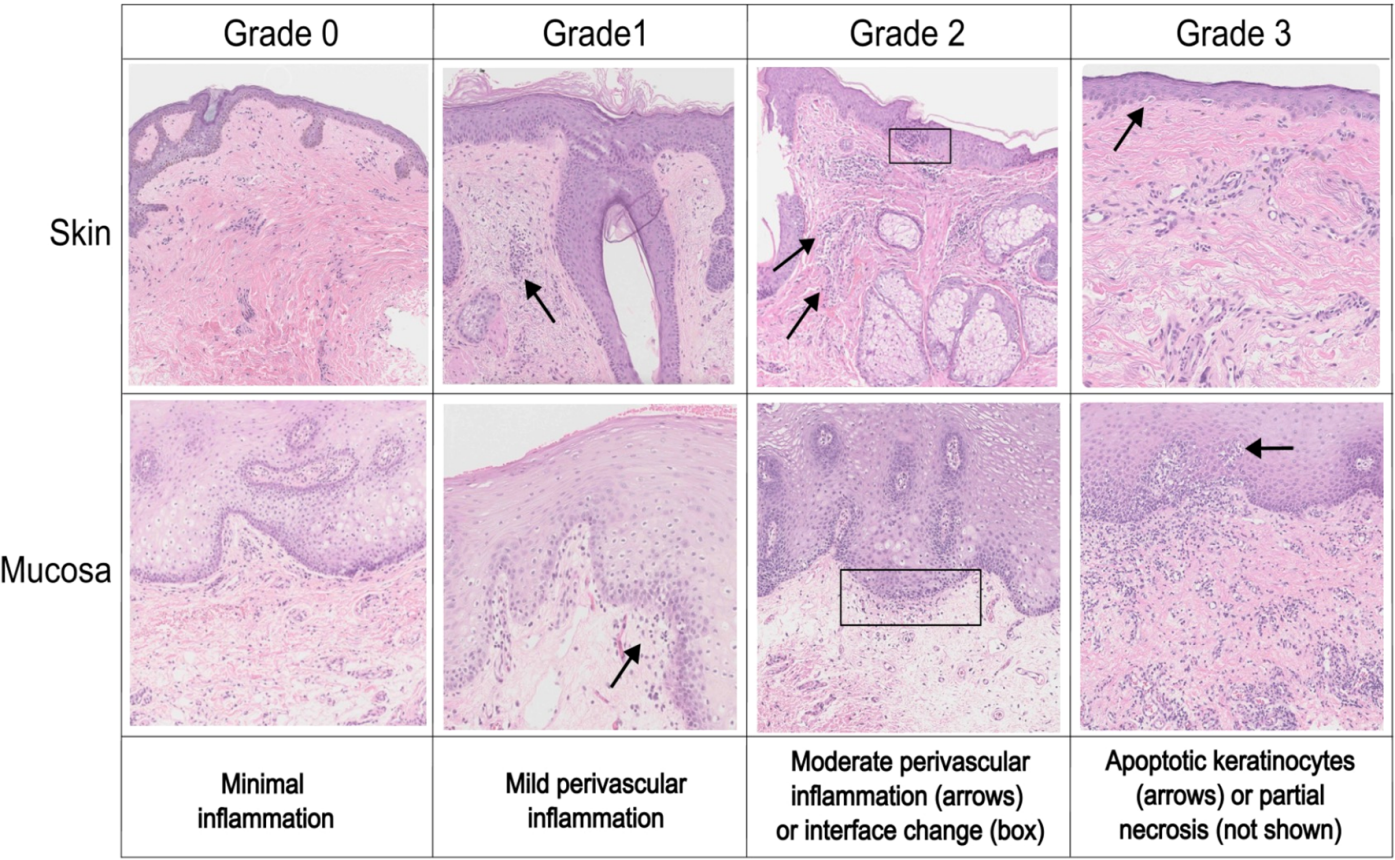
Representative histologic features of cutaneous and mucosal allograft rejection across Banff 2022 grades. The top row shows skin sections and the bottom row shows mucosal sections across rejection grades from 0 (non-rejection) to 3 (severe rejection). Grade 0 demonstrates preserved epithelial architecture without significant inflammatory infiltrates. Grade 1 shows mild superficial perivascular lymphocytic infiltrates without substantial epithelial involvement. Grade 2 is characterized by moderate inflammatory infiltrates and/or interface activity and early epithelial involvement, including lymphocytic exocytosis. Grade 3 demonstrates dense band-like inflammatory infiltrates with overt epithelial injury or (as shown here) more subtle apoptotic and dyskeratotic keratinocytes. All images are hematoxylin and eosin stained and are shown at variable magnification to emphasize diagnostic features. Grading is aligned with contemporary Banff 2022 criteria for acute rejection in vascularized composite allografts.

The 2007 classification grades acute cellular rejection by three features: epithelial lymphocytic infiltration; epidermal changes including spongiosis (intracellular edema) and keratinocyte apoptosis; and perivascular inflammation with or without lymphocytic exocytosis into the epithelium or pilosebaceous units (**Figure 1**). Standard 4-mm punch biopsies are H&E-stained. Grade 0 shows nonspecific changes with minimal inflammation; grade 1, mild superficial perivascular infiltrate without epidermal involvement; grade 2, moderate infiltrate with lymphocytic exocytosis or spongiosis but no keratinocyte necrosis; grade 3, dense band-like inflammation at the dermal-epidermal junction with keratinocyte apoptosis, dyskeratosis, or keratinolysis (1, 8). Grade 4 is frank epidermal necrosis.

## Mucosal and sentinel flap biopsies

Facial transplantation (fVCA) uniquely includes mucosa as an epithelial surface, increasingly recognized as a primary target of rejection. Unlike limb transplants, which rely solely on skin for surveillance, fVCA permits monitoring of both skin and mucosa. The Banff system does not yet define mucosal criteria, although several groups have proposed adaptations (9). Histologic features parallel those in skin: grade 0 with rare lymphocytes, grade 1 with perivascular infiltration, grade 2 with interface lymphocytic mucositis (± spongiosis), grade 3 with keratinocyte apoptosis, and grade 4 with necrosis (4, 5, 9, 10).

Our group and others have confirmed that cytotoxic T cells contribute to rejection in both skin and mucosa, with B-cell infiltration observed in mucosa, the relevance of which remains under investigation (5). Early studies suggest that mucosal inflammation may precede or persist longer than cutaneous changes (4). The oral mucosa heals rapidly and with minimal scarring because of accelerated epithelial repair and reduced fibrosis (11–13). Several centers have incorporated mucosal biopsies into routine surveillance. However, mucosal grading criteria are extrapolated from skin and can be confounded by mechanical trauma or infection; therefore, concurrent skin biopsies remain useful for excluding false−positive mucosal results and for assessing chronic dermal changes.

Fasciocutaneous sentinel flaps, such as donor-derived radial forearm flaps, are transplanted to a remote site during VCA placement to provide extra tissue that can be clinically monitored and biopsied to assess rejection (14, 15). Findings from mucosa and sentinel flaps suggest that assessing multiple tissue sites can better capture graft immunopathology than skin alone.

## Banff 2022 revision: addressing limitations and vascular changes

The Banff 2007 system focused on acute cellular rejection and excluded antibody-mediated and mucosal forms, which remain incompletely defined. Long-term experience revealed vascular inflammation and remodeling as important correlates of both acute and chronic VCA pathology. In recognition of this, the 2022 Banff working group revised the original 2007 framework to incorporate vascular injury and chronic changes that earlier criteria did not capture (7).

To address these gaps, the 2022 Banff introduced vascular modifiers to accompany the acute T cell-mediated rejection grade (7). Vasculitis (v0-v3) refers to mononuclear cells beneath the endothelium, graded by the extent of intimal inflammation or necrosis. Allograft vasculopathy (av0-av2) measures intimal thickening and luminal narrowing, and capillary thrombosis (t0-t1) notes the presence or absence of small-vessel thrombosis. These modifiers (for example, grade III, v2) extend the system to convey both the severity and chronicity of vascular injury.

Because these lesions often involve deep dermal or subcutaneous vessels, they may be missed in standard 4-mm punch biopsies. The group therefore recommended deeper sampling and complementary imaging. The revision also recognized vascular narrowing, arteritis, and microvessel thrombosis as hallmarks of severe acute rejection and possible indicators of chronic injury. Consistent with this update, Lian et al. examined a large series of facial graft biopsies and confirmed that T lymphocytes dominate the infiltrate during rejection (16), targeting microvessels and the stem cell-rich compartments of the epidermis and pilosebaceous units.

## Chronic rejection and long-term monitoring

Chronic rejection in VCA is not yet formally defined in the Banff classification but is recognized as a progressive, immune-mediated process that leads to structural and functional graft deterioration. It manifests through graft vasculopathy, dermal sclerosis, adnexal atrophy, and fibrosis (7, 17–19). Histologic features include intimal hyperplasia, vascular occlusion, band-like lymphohistiocytic infiltrates, loss of rete ridges, adnexal loss, and increased dermal collagen deposition (7, 17–19). At the molecular level, chronic rejection involves persistent activation of cytotoxic T cells and macrophages, upregulation of inflammatory cytokines such as IFN-γ, CXC chemokines CXCL9-11, and AP-1 pathway components *JUNB* and *FOS*, and activation of pathways such as IDO1 and STING (3, 19–22). These immune and metabolic pathways parallel those identified in acute facial allograft rejection, where IFN-γ and IDO1 signaling accompany cytotoxic T cell activation (23).

Diagnosis of chronic rejection depends on integrating clinical progression with histologic and vascular features, but reliable diagnostic thresholds remain undefined. Because deep arteries and subcutaneous vessels are often affected, standard 4-mm biopsies may miss these changes, so deeper sampling and imaging may improve detection. Persistent challenges include overlap with acute rejection, lack of validated biomarkers, and limited understanding of the transition to chronic disease (7, 17, 18, 21, 24).

## Comparative evaluation of tissue sites and sampling modalities

Given the limitations in current histologic sampling, several studies have compared the diagnostic performance of different tissue sites in VCA. Because skin is both visible and highly immunogenic, it has been used as the primary tissue for histopathologic evaluation of acute cellular rejection in VCA (24–27). Recent evidence indicates that skin may not always accurately reflect rejection occurring in deeper or other composite tissues, such as mucosa or muscle (**Table 1**). Studies in fVCA have shown that mucosal rejection can occur independently or with greater severity than skin rejection, and that exclusive reliance on skin biopsies may not detect important immune events in other graft components (2, 4, 5). However, direct correlations between mucosal inflammation and rejection in deeper graft compartments such as muscle or large vessels remain limited. Whether mucosal changes predict deeper tissue injury is an area of ongoing investigation. In a hand-transplant series, severe arterial intimal thickening and muscle fibrosis caused graft loss even when skin biopsies were Banff 0-1 (26, 28).

**Table 1:** Diagnostic performance of tissue modalities.

Modality (index → reference)	Sensitivity	Specificity	PPV	NPV	Clinical context	Key findings
Facial skin biopsy → Mucosal rejection	0.63	0.85	0.85	0.63	Both surveillance and for-cause (same paired-sampling framework).	Lower sensitivity vs. mucosa; skin rarely positive without mucosal involvement (3 encounters, often clinically non-relevant).
Mucosal biopsy → Facial skin rejection	0.85	0.63	0.63	0.85	Both surveillance and for-cause (serial 2–4 mm punch biopsies during suspected rejection or regular follow-up).	Strong correlation with skin (r≈0.72); mucosa captured rejection missed by skin in 10 encounters; ROC AUC ≈0.74. Supports integration into routine monitoring.
Sentinel flap biopsy → Facial skin rejection	0.75	0.94	0.94	0.76	Both surveillance and for-cause (paired sentinel–facial skin encounters).	High specificity/PPV but moderate sensitivity/NPV; missed ~25% of skin rejection episodes; ROC AUC ≈0.85; correlation r≈0.68. Not reliable as a substitute for facial skin biopsies.

Synthesis of diagnostic performance of the different tissue modalities in VCA. Mucosa often shows higher grades and stronger NPV (≈0.85) vs. skin; sentinel correlates best at higher rejection grades but misses events, favoring a multimodal approach. PPV, positive predictive value; NPV, negative predictive value; ROC, receiver-operating characteristic. Values derived from (22).

Each modality carries its own diagnostic trade-offs. Facial skin biopsy is accessible, well-validated, and supported by established Banff criteria, but is invasive, may scar, and is subject to sampling bias when rejection is patchy in the allograft. Mucosal biopsy provides an additional epithelial tissue that can be easily biopsied, though can be more challenging than the facial skin itself (4). Mucosa may detect early or discordant rejection in fVCA but is also subject to sampling bias given the patchy and discontinuous nature of mucosal involvement, and grading criteria are extrapolated from skin, and findings can be confounded by mechanical trauma or local infection (e.g. CMV) (2). Thus, mucosa may be seen as a supplement to skin biopsy, and its inherent value and biology need to be further validated and better understood.

Sentinel flaps allow biopsy from a remote site without disfiguring the primary graft and capture moderate-to-severe rejection reasonably well but show reduced discrimination of low-grade changes and may not always mirror events in the index allograft (2).

In a comparative analysis of 21 acute rejection episodes, mucosal and skin rejection grades were strongly correlated (r = 0.72), with mucosa demonstrating a high negative predictive value (≈0.85) and detecting several rejection episodes missed by skin (**Table 1**) (2). Sentinel flaps, such as radial forearm flaps, correlated with facial skin but showed lower mean grades and a lower NPV (~0.76), missing about 24 percent of cases (2). In a recent paired analysis of 14 simultaneous facial allograft and sentinel flap biopsies, 9 pairs showed identical Banff grades, with discordance in 5 encounters. Sentinel flaps tracked moderate-severe rejection well but showed reduced discrimination of grade 1 changes from non-rejection, while sharing grade 3 rejection-associated cytotoxic transcriptional programs and T cell clones with the facial graft (29). These findings suggest that skin biopsies are sensitive for acute cell mediated rejection but may miss deeper or parallel tissue injury, and they support a multimodal approach that includes mucosal and deeper sampling for more accurate detection and management of rejection, while recognizing that each modality has its own diagnostic limitations that warrant further calidation.

## Biomarkers and multimodal approaches

Serologic and immunohistochemical markers are being incorporated into monitoring strategies. In a cohort of nine fVCA recipients, donor−specific antibodies (DSAs) developed in 5 patients (30). Acute rejection grades tended to be higher when DSAs and C4d deposition were simultaneously positive, but chronic rejection occurred in two DSA−positive and two DSA−negative patients and only one graft was lost. Thus, DSAs have limited predictive value when used alone. Other adjuncts such as gene−expression profiling, circulating cytokines (e.g., *MMP3*), infrared thermography, and high−frequency ultrasound are being explored, but their diagnostic accuracy remains under investigation (20, 31, 32). Evidence suggests that a multimodal strategy combining clinical assessment, mucosal and skin biopsies, and immunologic biomarkers may improve detection of both acute and chronic rejection in face and limb VCA and may help tailor immunosuppression (5, 33).

## Molecular and biomarker approaches

Molecular diagnostics established in solid organ transplantation offer promising tools for VCA rejection monitoring. Circulating donor-derived cell-free DNA (dd-cfDNA) serves as a marker of allograft injury and accurately detects acute rejection across heart, kidney, and lung transplants. Seminal studies showed that elevated dd-cfDNA levels correlate with rejection episodes and often appear before clinical or histologic evidence (34–37). Recent multicenter studies link dd-cfDNA dynamics with antibody-mediated rejection and outcomes in kidney transplantation, which shows its early diagnostic sensitivity (38–40). These findings demonstrate dd-cfDNA’s translational potential for VCA, where early, minimally invasive monitoring could improve long-term graft outcomes (32, 41, 42). However, dd-cfDNA is a marker of allograft injury rather than rejection alone and cannot distinguish rejection from infection, ischemia, or other tissue injury. In VCA, interpretation may also be influenced by variability in graft size and tissue composition, and validated thresholds and prospective performance data remain undefined. Prospective validation in VCA is needed.

MicroRNAs (miRNAs) are emerging as promising biomarkers across kidney, liver, and VCA transplantation. Circulating signatures such as miR-155, miR-146a, miR-21-5p, miR-340-5p, miR-145-5p, and miR-195-5p distinguish tolerance from rejection, and antisense oligonucleotide therapies targeting these miRNAs are in development (43–45). Serum cytokines and chemokines, including *CXCL9*, *CXCL10*, *CXCL11*, and *IL6*, indicate immune activation and correlate with both acute and chronic rejection (20, 23, 46). High-throughput proteomic platforms such as Olink can detect these cytokine networks and broader protein injury signatures, though validation for VCA is in progress (41).

Minimally invasive approaches such as skin tape-strip assays and mucosal swabs permit serial analysis of gene and protein expression, reveal local immune responses, and may detect rejection events missed by conventional skin-focused methods (5). In fVCA, mucosal sampling could be particularly informative, as distinct gene-expression profiles can signal rejection patterns not seen in skin (5).

Tissue-based molecular assays, including gene-expression panels, spatial transcriptomics, and T-cell receptor (TCR) sequencing, provide high-resolution characterization of immune cell infiltration and activation. Gene-expression profiling platforms such as NanoString and the Banff Molecular Diagnostics Working Group panels have reclassified ambiguous histology in renal and cardiac transplants and identified rejection subtypes not detected by standard pathology (47, 48). Spatial transcriptomic platforms such as GeoMx provide multiplex gene-expression data while preserving tissue architecture, which reveals immune microenvironments during rejection (49). Integration of molecular data with histology and clinical findings increases diagnostic precision and supports personalized immunosuppression, reducing reliance on invasive biopsies (21, 32). These minimally invasive, multi-omic approaches have the potential to provide unprecedented diagnostic sensitivity and may transform rejection detection and management in VCA.

## Emerging and adjunctive modalities

Emerging and adjunctive modalities aim to address the limitations of biopsy-based rejection monitoring in VCA. Imaging techniques such as laser Doppler flowmetry, infrared thermography, and indocyanine green (ICG) angiography permit real-time, noninvasive evaluation of superficial perfusion and lymphatic drainage. Laser Doppler flowmetry measures microvascular blood flow and remains the standard for superficial tissue perfusion, though its depth is limited (50). Infrared thermography identifies early temperature changes that precede clinical symptoms and performs reliably in pigmented skin, offering a contact-free, objective complement to biopsy (31). ICG angiography and near-infrared lymphography visualize lymphatic drainage and contractility in VCA and permit assessment of lymphatic reconstitution and dysfunction (51). These methods cannot replace biopsy but may serve as adjuncts to improve early detection (32). Flow-based MRI quantifies vascular flow and arterial diameter to detect early graft vasculopathy before structural damage; however, it primarily reveals hemodynamic rather than immune changes and cannot distinguish rejection from infection or nonimmune injury (52).

Circulating biomarkers such as donor-derived cell-free DNA (dd-cfDNA) also hold promise for rejection monitoring in VCA. dd-cfDNA demonstrates high sensitivity and specificity for detecting graft injury in kidney, heart, and lung transplantation and is now used for noninvasive surveillance in clinical practice (41).

Digital pathology and artificial intelligence (AI) further strengthen histologic assessment. In solid organ transplantation, AI-assisted slide analysis reduces inter-observer variability, improves reproducibility, and supports pattern recognition across tissues (53). Recent kidney studies show that deep learning models quantify Banff features such as fibrosis and interstitial inflammation with high accuracy and distinguish borderline lesions from rejection (54). In VCA, similar methods could standardize Banff grading, harmonize multicenter studies, and provide objective measures of skin and mucosal pathology. Together, these modalities may supplement histology with minimally invasive, real-time data on perfusion, microvascular integrity, and tissue health and could support earlier, more precise intervention.

## Toward Integration and noninvasive diagnostic approaches

The high incidence of acute rejection in VCA and the limitations of relying on skin biopsy alone have fueled the development of less invasive, more comprehensive diagnostic strategies. A systematic review identified 17 distinct non- or minimally invasive monitoring modalities in both human and animal models for diagnosing or monitoring allograft rejection, spanning noninvasive vascular imaging, liquid biomarkers, epidermal sampling, clinical grading scales, and sentinel flaps (55–57).

Serologic and cellular biomarkers have shown promise as adjuncts in diagnosing acute rejection. In a 2019 multicenter study led by our group, serum MMP3 levels spiked during severe rejection in VCAs (58). One limitation of this early study was the lack of mucosal biopsies in fVCAs, leaving open how such levels might be influenced by rejection in other tissues. Similarly, a retrospective analysis of three face transplant recipients found that peripheral lymphocyte subsets (CD3^+^/CD4^+^ T cells and CD16^+^/CD56^+^ NK cells) were significantly upregulated during rejection (59). In another study using standard laboratory tests (CBC and differential, CRP, BMP), Kauke et al. observed that CRP increased during acute rejection episodes, which was corroborated in a small animal model (56, 57). A further study using CyTOF confirmed at the single-cell level the upregulation of several key immune cell types during rejection, including CD8^+^/GZMB^+^ T cells, with levels spiking significantly during combined skin and mucosal rejection (5).

In the future, these diagnostic adjuncts could complement routine histology to help identify molecular or cellular patterns that improve diagnostic accuracy, particularly given that several dermatoses can mimic rejection histologically (3). However, noninvasive monitoring alone remains unrealistic at the current stage, as none of these methods have been validated, and even changes in lymphocyte populations are not diagnostic of rejection. Indeed, in standard CBCs, total WBC and lymphocyte counts did not differ significantly between rejection and non-rejection episodes (57). The extent of systemic immune changes may depend on graft size, as smaller, partial fVCAs may not elicit measurable systemic shifts.

Novel molecular markers may eventually supplement histologic assessment, such as transcriptomic profiling similar to the Banff Human Organ Transplant (B-HOT) gene panel being explored in kidney transplant diagnostics (48). However, due to the small number of VCAs, biopsy of the skin (+mucosa for fVCAs) remain the most commonly used diagnostic approaches in routine clinical practice (2). Incorporating vascular involvement scoring into the Banff classification represents progress toward a more comprehensive framework, yet the biology of rejection is not fully defined.

Moving forward, standardization and collaboration across transplant centers will be essential to unify diagnostic approaches and refine management strategies aimed at early detection and prevention of chronic rejection. Detailed analysis of paired skin and mucosal biopsy data (for fVCA) represents an important first step. Collaboration among leading centers to validate and implement novel diagnostic tools through registries, pooled data analysis, and open discourse will accelerate translation into routine practice and improve early rejection detection and long-term graft outcomes.

## Discussion

As diagnostic technologies expand beyond histology, integrating these modalities into a unified framework will require practical workflows that connect tissue-based and noninvasive data streams. Routine skin and mucosal biopsies could continue to anchor histologic grading, while parallel assays such as dd-cfDNA, serum cytokine panels, and transcriptomic profiling could provide quantitative biomarkers of rejection activity. Imaging modalities, including infrared thermography and ICG angiography, offer immediate functional assessment of perfusion that can guide biopsy targeting and longitudinal monitoring. Combining these data within standardized scoring systems or machine learning pipelines could support objective, reproducible diagnosis across centers and reduce reliance on histology alone.

Skin remains the most accessible and validated tissue for diagnosing rejection in VCA, but it may not always reflect immune injury in deeper or parallel tissues. Mucosal rejection can occur independently or with greater severity than cutaneous rejection, meaning skin biopsies alone may overlook key immune events. Debate persists over whether mucosal sampling should supplant or complement skin biopsy for routine surveillance. The Banff 2022 revision addressed limitations of the 2007 framework by adding vascular modifiers that capture vasculitis and chronic vascular changes, yet standardized criteria for antibody-mediated and chronic rejection remain limited. Advancing VCA diagnostics will require collective effort: multicenter collaboration, shared data infrastructure, and harmonized criteria that move the field from experimental to standardized clinical practice. The addition of mucosal biopsies and assessment may be a valuable addition to the holistic assessment of immune activity in a facial allograft.

## References

[1] Cendales LC Kanitakis J Schneeberger S Burns C Ruiz P Landin L . The Banff 2007 working classification of skin-containing composite tissue allograft pathology. Am J Transplant. (2008) 8:1396–400. doi: 10.1111/j.1600-6143.2008.02243.x. PMID: 18444912

[2] Kauke-Navarro M Huelsboemer L Klimitz FJ Diatta F Knoedler L Knoedler S . A comparative analysis of lesional skin, sentinel flap, and mucosal biopsies in assessing acute face transplant rejection. Front Immunol. (2025) 16:1562024. doi: 10.3389/fimmu.2025.1562024. PMID: 40236712 PMC11997448

[3] Knoedler L Knoedler S Panayi AC Lee CAA Sadigh S Huelsboemer L . Cellular activation pathways and interaction networks in vascularized composite allotransplantation. Front Immunol. (2023) 14:1179355. doi: 10.3389/fimmu.2023.1179355. PMID: 37266446 PMC10230044

[4] Kauke M Safi AF Zhegibe A Haug V Kollar B Nelms L . Mucosa and rejection in facial vascularized composite allotransplantation: A systematic review. Transplantation. (2020) 104:2616–24. doi: 10.1097/tp.0000000000003171. PMID: 32053572

[5] Kauke-Navarro M Crisler WJ Younis N Khetani RS Sadigh S Teague JE . B-cell infiltration distinguishes mucosal from skin patterns of rejection in facial vascularized composite allografts. Am J Transplant. (2025) 25:1193–207. doi: 10.1016/j.ajt.2025.01.013. PMID: 39842779

[6] Haykal S Barone N Rostami S Moshkelgosha S Juvet S Keshavjee S . Immunohistochemical analysis of lymphocyte populations in acute skin rejection: The University Health Network addition to the Banff classification. Plast Reconstr Surg Glob Open. (2023) 11:e4831. doi: 10.1097/gox.0000000000004831. PMID: 36875922 PMC9984153

[7] Cendales LC Farris AB Rosales I Elder D Gamboa-Dominguez A Gelb B . Banff 2022 vascularized composite allotransplantation meeting report: Diagnostic criteria for vascular changes. Am J Transplant. (2024) 24:716–23. doi: 10.2139/ssrn.4449819 38286355

[8] Deo PN Deshmukh R . Pathophysiology of keratinization. J Oral Maxillofac Pathol. (2018) 22:86–91. doi: 10.4103/jomfp.jomfp_195_16. PMID: 29731562 PMC5917548

[9] Bergfeld W Klimczak A Stratton JS Siemionow MZ . A four-year pathology review of the near total face transplant. Am J Transplant. (2013) 13:2750–64. doi: 10.1111/ajt.12379. PMID: 23919328

[10] Kanitakis J Badet L Petruzzo P Beziat JL Morelon E Lefrancois N . Clinicopathologic monitoring of the skin and oral mucosa of the first human face allograft: Report on the first eight months. Transplantation. (2006) 82:1610–5. doi: 10.1097/01.tp.0000248780.55263.33. PMID: 17198245

[11] Glim JE van Egmond M Niessen FB Everts V Beelen RH . Detrimental dermal wound healing: what can we learn from the oral mucosa? Wound Repair Regener. (2013) 21:648–60. doi: 10.1111/wrr.12072. PMID: 23927738

[12] Pereira D Sequeira I . A scarless healing tale: Comparing homeostasis and wound healing of oral mucosa with skin and oesophagus. Front Cell Dev Biol. (2021) 9:682143. doi: 10.3389/fcell.2021.682143. PMID: 34381771 PMC8350526

[13] Waasdorp M Krom BP Bikker FJ van Zuijlen PPM Niessen FB Gibbs S . The bigger picture: Why oral mucosa heals better than skin. Biomolecules. (2021) 11:1165. doi: 10.3390/biom11081165. PMID: 34439831 PMC8394648

[14] Kueckelhaus M Fischer S Lian CG Bueno EM Marty FM Tullius SG . Utility of sentinel flaps in assessing facial allograft rejection. Plast Reconstr Surg. (2015) 135:250–8. doi: 10.1097/prs.0000000000000797. PMID: 25255116

[15] Petruzzo P Testelin S Kanitakis J Badet L Lengele B Girbon JP . First human face transplantation: 5 years outcomes. Transplantation. (2012) 93:236–40. doi: 10.1097/TP.0b013e31823d4af6 22167048

[16] Lian CG Bueno EM Granter SR Laga AC Saavedra AP Lin WM . Biomarker evaluation of face transplant rejection: association of donor T cells with target cell injury. Mod Pathol. (2014) 27:788–99. doi: 10.1038/modpathol.2013.249. PMID: 24434898

[17] Brandacher G Messner F Al-Salmay Y Kaufman CL . Clinical manifestation, mechanisms, and potential targets of intervention for chronic rejection in vascularized composite allotransplantation. Transplantation. (2025) 109:1710–22. doi: 10.1097/tp.0000000000005450. PMID: 40626707

[18] Kanitakis J Petruzzo P Badet L Gazarian A Thaunat O Testelin S . Chronic rejection in human vascularized composite allotransplantation (Hand and face recipients): An update. Transplantation. (2016) 100:2053–61. doi: 10.1097/mot.0000000000000571. PMID: 27163543

[19] Krezdorn N Lian CG Wells M Wo L Tasigiorgos S Xu S . Chronic rejection of human face allografts. Am J Transplant. (2019) 19:1168–77. doi: 10.1111/ajt.15143. PMID: 30312535 PMC6433509

[20] Puscz F Dadras M Dermietzel A Jacobsen F Lehnhardt M Behr B . A chronic rejection model and potential biomarkers for vascularized composite allotransplantation. PloS One. (2020) 15:e0235266. doi: 10.1371/journal.pone.0235266. PMID: 32589662 PMC7319338

[21] Thomé J Lind M Schmitt M Schneider L Kiefer J Schäfer R . Differentiation of acute versus chronic skin rejection in a rodent model of vascularized composite allotransplantation. Front Immunol. (2025) 16:1672754. doi: 10.3389/fimmu.2025.1672754 41098717 PMC12518069

[22] Lee CAA Wang D Kauke-Navarro M Russell-Goldman E Xu S Mucciarone KN . Insights from immunoproteomic profiling of a rejected full-face transplant. Am J Transplant. (2023) 23:1058–61. doi: 10.1016/j.ajt.2023.04.008. PMID: 37037378

[23] Win TS Crisler WJ Dyring-Andersen B Lopdrup R Teague JE Zhan Q . Immunoregulatory and lipid presentation pathways are upregulated in human face transplant rejection. J Clin Invest. (2021) 131:e135166. doi: 10.1172/jci135166. PMID: 33667197 PMC8262560

[24] Sarhane KA Tuffaha SH Broyles JM Ibrahim AE Khalifian S Baltodano P . A critical analysis of rejection in vascularized composite allotransplantation: clinical, cellular and molecular aspects, current challenges, and novel concepts. Front Immunol. (2013) 4:406. doi: 10.3389/fimmu.2013.00406. PMID: 24324470 PMC3839257

[25] Morelon E Kanitakis J Petruzzo P . Immunological issues in clinical composite tissue allotransplantation: where do we stand today? Transplantation. (2012) 93:855–9. doi: 10.1097/TP.0b013e31824728b8 22538449

[26] Robbins NL Wordsworth MJ Parida BK Kaplan B Gorantla VS Weitzel EK . Is skin the most allogenic tissue in vascularized composite allotransplantation and a valid monitor of the deeper tissues? Plast Reconstr Surg. (2019) 143:880e–6e. doi: 10.1097/prs.0000000000005436. PMID: 30921156

[27] Kanitakis J Petruzzo P Gazarian A Karayannopoulou G Buron F Dubois V . Capillary thrombosis in the skin: A pathologic hallmark of severe/chronic rejection of human vascularized composite tissue allografts? Transplantation. (2016) 100:954–7. doi: 10.1097/TP.0000000000000882 27003099

[28] Kaufman CL Ouseph R Blair B Kutz JE Tsai TM Scheker LR . Graft vasculopathy in clinical hand transplantation. Am J Transplant. (2012) 12:1004–16. doi: 10.1111/j.1600-6143.2011.03915.x. PMID: 22325051

[29] Crisler WJ Win TS Kauke-Navarro M Zhan Q Barrera V Sui SH . Sentinel flaps reflect key inflammatory aspects of human face transplant rejection. Am J Transplant. (2025) 26:1035–47. doi: 10.1016/j.ajt.2025.12.009. PMID: 41407105

[30] Huelsboemer L Hosseini H Klimitz FJ Fortunay D Boroumand S O'Brien C . The prognostic relevance of donor-specific antibodies in facial transplantation – a retrospective cohort study. J Plast Reconstr Aesthet Surg. (2025) 103:286–96. doi: 10.1016/j.bjps.2025.01.028. PMID: 40043533

[31] Filz von Reiterdank I Jain R de Clermont-Tonnerre E Tchir A Cetrulo CL Lellouch AG . Thermal rejection assessment: New strategies for early detection. Transpl Int. (2025) 38:14108. doi: 10.1016/j.ajt.2025.07.692. PMID: 40309263 PMC12040617

[32] Honeyman C Stark H Wang HC Hester J Issa F Giele H . Biomarker and surrogate development in vascularised composite allograft transplantation: Current progress and future challenges. J Plast Reconstr Aesthet Surg. (2021) 74:711–7. doi: 10.1016/j.bjps.2020.11.022. PMID: 33436335

[33] Kueckelhaus M Fischer S Seyda M Bueno EM Aycart MA Alhefzi M . Vascularized composite allotransplantation: current standards and novel approaches to prevent acute rejection and chronic allograft deterioration. Transpl Int. (2016) 29:655–62. doi: 10.1111/tri.12652. PMID: 26265179 PMC4785085

[34] De Vlaminck I Valantine HA Snyder TM Strehl C Cohen G Luikart H . Circulating cell-free DNA enables noninvasive diagnosis of heart transplant rejection. Sci Transl Med. (2014) 6:241ra77. doi: 10.1126/scitranslmed.3007803. PMID: 24944192 PMC4326260

[35] Snyder TM Khush KK Valantine HA Quake SR . Universal noninvasive detection of solid organ transplant rejection. Proc Natl Acad Sci USA. (2011) 108:6229–34. doi: 10.1073/pnas.1013924108. PMID: 21444804 PMC3076856

[36] García Moreira V Prieto García B Baltar Martín JM Ortega Suárez F Alvarez FV . Cell-free DNA as a noninvasive acute rejection marker in renal transplantation. Clin Chem. (2009) 55:1958–66. doi: 10.1373/clinchem.2009.129072. PMID: 19729469

[37] Bloom RD Bromberg JS Poggio ED Bunnapradist S Langone AJ Sood P . Cell-free DNA and active rejection in kidney allografts. J Am Soc Nephrol. (2017) 28:2221–32. doi: 10.1681/asn.2016091034. PMID: 28280140 PMC5491290

[38] Aubert O Ursule-Dufait C Brousse R Gueguen J Racapé M Raynaud M . Cell-free DNA for the detection of kidney allograft rejection. Nat Med. (2024) 30:2320–7. doi: 10.1038/s41591-024-03087-3. PMID: 38824959 PMC11333280

[39] Akifova A Budde K Amann K Buettner-Herold M Choi M Oellerich M . Donor-derived cell-free DNA monitoring for early diagnosis of antibody-mediated rejection after kidney transplantation: a randomized trial. Nephrol Dial Transplant. (2025) 40:1384–95. doi: 10.1093/ndt/gfae282. PMID: 39673311 PMC12207606

[40] Bunnapradist S Leca N Zaky ZS Stratta RJ Khamash HA Shihab F . Associations between donor-derived cell-free DNA dynamics and clinical outcomes after kidney allograft rejection: A prospective, multicenter study. Am J Transplant. (2025) 25:2543–53. doi: 10.1016/j.ajt.2025.07.2470. PMID: 40712889

[41] Song Y Wang Y Wang W Xie Y Zhang J Liu J . Advancements in noninvasive techniques for transplant rejection: from biomarker detection to molecular imaging. J Transl Med. (2025) 23:147. doi: 10.1186/s12967-024-05964-4. PMID: 39901268 PMC11792214

[42] Jimenez-Coll V Llorente S Boix F Alfaro R Galián JA Martinez-Banaclocha H . Monitoring of serological, cellular and genomic biomarkers in transplantation, computational prediction models and role of cell-free DNA in transplant outcome. Int J Mol Sci. (2023) 24:3908. doi: 10.3390/ijms24043908. PMID: 36835314 PMC9963702

[43] Anglicheau D Sharma VK Ding R Hummel A Snopkowski C Dadhania D . MicroRNA expression profiles predictive of human renal allograft status. Proc Natl Acad Sci USA. (2009) 106:5330–5. doi: 10.1073/pnas.0813121106. PMID: 19289845 PMC2663998

[44] Matz M Lorkowski C Fabritius K Durek P Wu K Rudolph B . Free microRNA levels in plasma distinguish T-cell mediated rejection from stable graft function after kidney transplantation. Transpl Immunol. (2016) 39:52–9. doi: 10.1016/j.trim.2016.09.001. PMID: 27663089

[45] Fang Y Li H Zhu Y Chen J Xiong Y Li X . Expression and predictive functional profiles of microRNAs data in vascularized composite allotransplantation acute rejection. Front Biosci (Landmark Ed). (2022) 27:279. doi: 10.31083/j.fbl2710279. PMID: 36336858

[46] Tharmaraj D Mulley WR Dendle C . Current and emerging tools for simultaneous assessment of infection and rejection risk in transplantation. Front Immunol. (2024) 15:1490472. doi: 10.3389/fimmu.2024.1490472. PMID: 39660122 PMC11628869

[47] Zhang H Haun RS Collin F Cassol C Napier JOH Wilson J . Development and validation of a multiclass model defining molecular archetypes of kidney transplant rejection: A large cohort study of the Banff human organ transplant gene expression panel. Lab Invest. (2024) 104:100304. doi: 10.1016/j.labinv.2023.100304. PMID: 38092179

[48] Mengel M Loupy A Haas M Roufosse C Naesens M Akalin E . Banff 2019 meeting report: Molecular diagnostics in solid organ transplantation-consensus for the Banff human organ transplant (B-HOT) gene panel and open source multicenter validation. Am J Transplant. (2020) 20:2305–17. doi: 10.1111/ajt.16059. PMID: 32428337 PMC7496585

[49] Ton C Salehi S Abasi S Aggas JR Liu R Brandacher G . Methods of ex vivo analysis of tissue status in vascularized composite allografts. J Transl Med. (2023) 21:609. doi: 10.1186/s12967-023-04379-x. PMID: 37684651 PMC10492401

[50] Misra S Shishehbor MH Takahashi EA Aronow HD Brewster LP Bunte MC . Perfusion assessment in critical limb ischemia: Principles for understanding and the development of evidence and evaluation of devices: A scientific statement from the American Heart Association. Circulation. (2019) 140:e657–72. doi: 10.1161/cir.0000000000000708. PMID: 31401843 PMC7372288

[51] Buretta KJ Brat GA Christensen JM Ibrahim Z Grahammer J Furtmüller GJ . Near-infrared lymphography as a minimally invasive modality for imaging lymphatic reconstitution in a rat orthotopic hind limb transplantation model. Transpl Int. (2013) 26:928–37. doi: 10.1111/tri.12150. PMID: 23879384

[52] Bettoni J Balédent O Petruzzo P Duisit J Kanitakis J Devauchelle B . Role of flow magnetic resonance imaging in the monitoring of facial allotransplantations: preliminary results on graft vasculopathy. Int J Oral Maxillofac Surg. (2020) 49:169–75. doi: 10.1016/j.ijom.2019.05.003. PMID: 31235388

[53] Rahman MA Yilmaz I Albadri ST Salem FE Dangott BJ Taner CB . Artificial intelligence advances in transplant pathology. Bioengineering (Basel). (2023) 10:1041. doi: 10.3390/bioengineering10091041. PMID: 37760142 PMC10525684

[54] Fayzullin A Ivanova E Grinin V Ermilov D Solovyeva S Balyasin M . Towards accurate and efficient diagnoses in nephropathology: An AI-based approach for assessing kidney transplant rejection. Comput Struct Biotechnol J. (2024) 24:571–82. doi: 10.1016/j.csbj.2024.08.011. PMID: 39258238 PMC11385065

[55] Stead TS Brydges HT Laspro M Onuh OC Chaya BF Rabbani PS . Minimally and non-invasive approaches to rejection identification in vascularized composite allotransplantation. Transplant Rev (Orlando). (2023) 37:100790. doi: 10.1016/j.trre.2023.100790. PMID: 37625211

[56] Kiefer J Zeller J Schneider L Thome J McFadyen JD Hoerbrand IA . C-reactive protein orchestrates acute allograft rejection in vascularized composite allotransplantation via selective activation of monocyte subsets. J Adv Res. (2025) 72:401–20. doi: 10.1016/j.jare.2024.07.007. PMID: 38992424 PMC12147648

[57] Kauke-Navarro M Knoedler S Panayi AC Knoedler L Haller B Parikh N . Correlation between facial vascularized composite allotransplantation rejection and laboratory markers: Insights from a retrospective study of eight patients. J Plast Reconstr Aesthet Surg. (2023) 83:155–64. doi: 10.1016/j.bjps.2023.04.050. PMID: 37276734

[58] Kollar B Uffing A Borges TJ Shubin AV Aoyama BT Dagot C . MMP3 is a non-invasive biomarker of rejection in skin-bearing vascularized composite allotransplantation: A multicenter validation study. Front Immunol. (2019) 10:2771. doi: 10.3389/fimmu.2019.02771. PMID: 31849957 PMC6897344

[59] Chinta SR Shah AR Tran DL Lee WY Mangiola M Gelb BE . New paradigms in rejection monitoring: Lymphocyte subsets as noninvasive graft markers in vascularized composite allotransplantation. Plast Reconstr Surg Glob Open. (2025) 13:e6598. doi: 10.1097/gox.0000000000006598. PMID: 40051973 PMC11884835

